# COMMUNITY-ENGAGED AGING RESEARCH IN GHANA

**DOI:** 10.1093/geroni/igae098.1873

**Published:** 2024-12-31

**Authors:** Tiffany Washington, Fayron Epps

**Affiliations:** Chair; Discussant

## Abstract

Sub-Saharan Africa (SSA) countries have the fastest growing rates of older adults than other countries across the globe. In SSA countries, adults aged 60 and older will reach 161 million by 2050. Because SSA countries have under-developed economies, older adults in this region are at greater risk for a myriad of social, economic, and health conditions in comparison to their developed counterparts. For example, estimates suggest that the prevalence of dementia in the SSA region may be considerably higher than previously assumed, given the scarcity of comprehensive epidemiological data and the rapidly aging population. Addressing this knowledge gap is imperative to develop effective strategies for early detection, intervention, and support for affected individuals and their families. Therefore, the goal of this symposium is to describe efforts to better understand the experience of older adults in Ghana through community-university partnerships. Speaker one will discuss the development and implementation of a culturally adapted dementia education intervention in Ghana. Speaker two will describe nursing students’ experiences caring for older adults in acute care settings in Ghana. Speaker three will present barriers and facilitators to global community engaged dementia research using a collaboration between U.S.-based university and Ghanaian community partners as a case example. The discussant will outline steps forward in community-engaged aging research in Ghana.

